# Supplementary material for: The interactome of intact mitochondria by cross-linking mass spectrometry provides evidence for coexisting respiratory supercomplexes
Source: Mol Cell Proteomics. 2017 Dec 8;17(2):216–32. doi: 10.1074/mcp.RA117.000470 (PMC5795388; doi:10.1074/mcp.RA117.000470)
Supplement: Supplemental Data [file supp_RA117.000470_133922_0_supp_23978_fzffwf.zip › spectra_annotation/mito_DR_spectra_annotation/198-2-3-1-116-1.pdf]

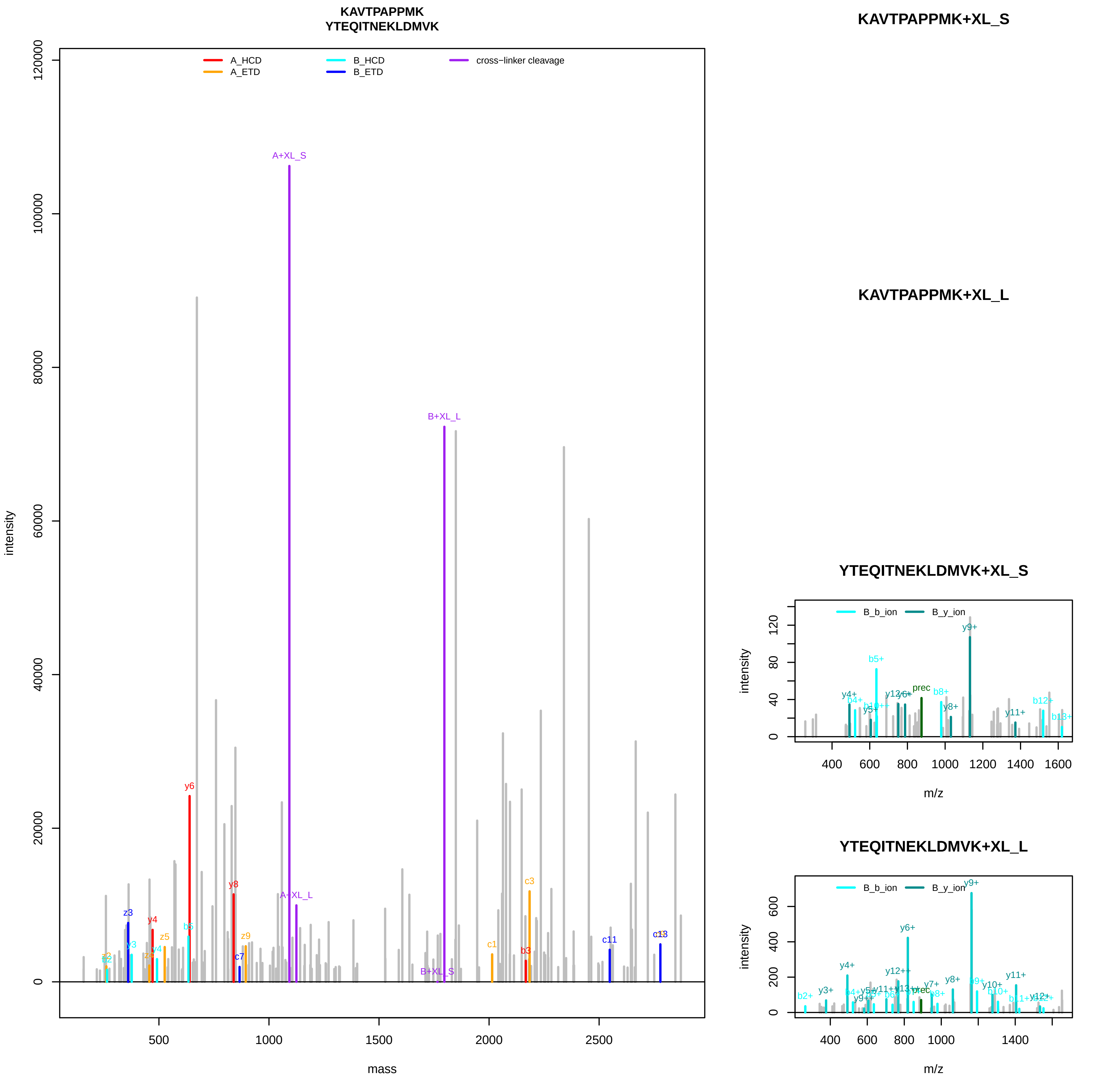
KAVTPAPPMK
YTEQITNEKLDMVK
A_HCD
A_ETD
B_HCD
B_ETD
cross−linker cleavage
A+XL_S
B+XL_L
A+XL_L
B+XL_S
y6
y8
y4
b3
c3
c1
z3
z5
z9
c7
c11
c13
intensity
mass
KAVTPAPPMK+XL_S
KAVTPAPPMK+XL_L
YTEQITNEKLDMVK+XL_S
B_b_ion
B_y_ion
y9+
b5+
prec
b8+
y8+
y4+
b4+
y5+
y11+
b12+
b13+
m/z
YTEQITNEKLDMVK+XL_L
y6+
y12++
y10+
b10+
y7+
b2+
y3+
